# Monocular Visual-Inertial Odometry with an Unbiased Linear System Model and Robust Feature Tracking Front-End

**DOI:** 10.3390/s19081941

**Published:** 2019-04-25

**Authors:** Xiaochen Qiu, Hai Zhang, Wenxing Fu, Chenxu Zhao, Yanqiong Jin

**Affiliations:** 1 School of Automation Science and Electrical Engineering, Beihang University, No. 37 Xueyuan Road, Haidian District, Beijing 100191, China; qiuxiaochen@buaa.edu.cn (X.Q.); buaa_jinyq@buaa.edu.cn (Y.J.); 2 Science and Technology on Aircraft Control Laboratory, Beihang University, No. 37 Xueyuan Road, Haidian District, Beijing 100191, China; 3 Science and Technology on Complex System Control and Intelligent Agent Cooperation Laboratory, No. 40 Yungangbeili, Fengtai District, Beijing 100074, China; fuwenxingCHN@163.com; 4 Sino-French Engineer School, Beihang University, No. 37 Xueyuan Road, Haidian District, Beijing 100191, China; chenxu.zhao@buaa.edu.cn

**Keywords:** visual inertial odometry, quaternion notation, closed-form state transition equation, robust feature tracking, real-time motion tracking, computation saving

## Abstract

The research field of visual-inertial odometry has entered a mature stage in recent years. However, unneglectable problems still exist. Tradeoffs have to be made between high accuracy and low computation for users. In addition, notation confusion exists in quaternion descriptions of rotation; although not fatal, this may results in unnecessary difficulties in understanding for researchers. In this paper, we develop a visual-inertial odometry which gives consideration to both precision and computation. The proposed algorithm is a filter-based solution that utilizes the framework of the noted multi-state constraint Kalman filter. To dispel notation confusion, we deduced the error state transition equation from scratch, using the more cognitive Hamilton notation of quaternion. We further come up with a fully linear closed-form formulation that is readily implemented. As the filter-based back-end is vulnerable to feature matching outliers, a descriptor-assisted optical flow tracking front-end was developed to cope with the issue. This modification only requires negligible additional computation. In addition, an initialization procedure is implemented, which automatically selects static data to initialize the filter state. Evaluations of proposed methods were done on a public, real-world dataset, and comparisons were made with state-of-the-art solutions. The experimental results show that the proposed solution is comparable in precision and demonstrates higher computation efficiency compared to the state-of-the-art.

## 1. Introduction

The fusion of monocular cameras and inertial measurement units (IMUs) is very popular recently, thanks to great improvements in the computation capacity of computers with low energy costs and low weight, and the increasing demand for accurate motion tracking or positioning in unmanned aerial vehicles (UAVs), augmented reality, and driverless cars. This fusion problem has been studied by brilliant scientists for years [1,2,3,4], and as a result, one can now build his own visual-inertial odometry (VIO) module simply with cheap sensors and open-source software [2,5,6,7,8,9].

Fusion frameworks are divided into two main branches: filter-based and optimization- based [10,11]. Optimization-based methods are so far widely recognized performing better in terms of precision [12] due to their iterating mechanism, which is essentially solving a noted bundle adjustment (BA) problem [13]. The BA problem was considered to be computational costly in earlier years, until the literature recognized and revealed its sparse structure [13,14] so as to develop real-time algorithms. Kümmerle et al. [7] modeled BA as a graph optimization problem. Kaess et al. [8] introduced a factor graph model to further illustrate the Bayesian nature of BA. Kaess et al. [8] also found that the incremental fact of sophiscated Hessian matrix in normal equation can be utilized for solving BA, thus speeding up the calculation further. With these profound insights, researchers also made efforts to overcome the inconsistency in fixed-lag fusion algorithms, which has the advantages of having bounded computation with less information loss and maintaining the sparse structure [15]. Several open-source libraries are available for building the back-end for algorithms of this branch, based on different mathematical descriptions listed above and providing convenient application program interfaces (APIs) [7,8,9,16]. Although enabling reduced computation by leveraging sparse matrix factorization, optimization-based VIO systems still need to be tailored sometimes in order to be deployed on a computation-limited platform. Sometimes, this leads to a downgraded performance [17].

Before the flowering of optimization-based methods, the solving of fusion problems was dominated by filtering [18]. The ordinary procedure is to include IMU pose and map point positions in the filter state and recursively propagate and update as IMU and camera measurements respectively arrive [10]. The accurate estimation of map point positions is the key to bring about an unbiased IMU pose updating. In traditional filter-based solutions, the filter state would invariably have a very large dimension since it always preserves a lot of map points, resulting in enhanced computation requirements [1]. The use of a multi-state constraint Kalman filter (MSCKF) was proposed as an effective and optimal filter-based solution that does not maintain map points in filter state [19]. By properly handling the camera measurement, MSCKF can achieve as competitive a performance as optimization-based algorithms and demands far less computation [17].

By correcting observability properties [19,20,21] and incorporating camera–IMU extrinsic parameters into the filter state [22], the performance of MSCKF was further improved. Many follow-up works emerged, including an open-source monocular implementation [23], expansion to stereo camera rig [24], and schemes using direct visual front-ends [25] or adding line features [26].

It should be emphasized that all members of the MSCKF family so far have been developed based on Shuster’s notation of quaternion [27], whereas most of the community utilizes the traditional Hamilton notation, which results in unnecessary trouble in understanding for researchers [28].

Visual front-ends apparently play an important role in VIOs. There are typically of two categories. Feature-based methods use descriptors to match features between consecutive images [6], while direct methods seek a minimization of photometric residuals to accomplish data correlation [5,25]. Sparse optical flow tracking is an efficient direct method that is widely used [2,23,24]. It provides sub-pixel accuracy but contains more outliers than feature descriptor matching [29]. An optimization-based back-end would eliminate outliers during iteration [30]. Filter-based back-ends are meanwhile vulnerable to the outliers if only one-off updating is applied [23]. Using an iterated update scheme would mitigate this situation while introducing additive computation [31].

To recap, in order to make VIO algorithms more practical, it is desirable to develop algorithms with lower computation while maintaining high precision.

In this paper, we developed a filter-based monocular visual-inertial odometry which can be regarded as a member of MSCKF family, giving consideration to both high precision and computation efficiency. The main contributions of this paper are as follows:

- We deduced a closed-form IMU error state transition equation based on the more cognitive Hamilton notation of quaternion. By solving integration terms analytically, a novel fully linear formulation was further obtained, which is also closed-form, and furthermore, is readily implemented.
- By analyzing the statistical properties of ORB descriptor [32] distances of matched and unmatched feature points, we introduced a novel descriptor-assisted sparse optical flow tracking technique, which enhances the feature tracking robustness and barely adds any computation complexity.
- More improvements are made to improve the usability and performance of the filter. An initialization procedure is developed that automatically detects stationary scenes by analyzing tracked features and initializes the filter state based on static IMU data. The feature triangulation mechanism is carefully refined to provide efficient measurement updates.
- A filter-based monocular VIO using the proposed state transition equation, visual front-end, and initialization procedure under Sun et al.’s [24] framework is implemented. The performances of our VIO and MSCKF-MONO [23], an open-source monocular implementation of MSCKF, are compared with parameters setup as similarly as possible. Ours is also compared with other state-of-the-art open-source VIOs including ROVIO [5], OKVIS [6], and VINS-MONO [2]. In addition, we analyze the process time of our algorithm. All of the evaluations above are done on EuRoC datasets [33]. Detailed evaluations are reported.

The rest of this paper is organized as follows. The problem of quaternion notation confusion is illustrated in Section 2. Section 3 deduces the error state differential equation based on Hamilton’s notation. Section 4 gives a closed-form error state transition formulation and then solves the integration terms in it, obtaining a fully linear closed-form formulation. Section 5 presents the descriptor-assisted sparse optical flow tracking front-end. Other implementation details and improvements are presented in Section 6, including the overall filter model, automatic initialization procedure, and refined feature triangulation mechanism. Section 7 presents the experimental results in detail. Finally, conclusions are made in Section 8.

## 2. Quaternion Notation Confusion

Quaternion is one of the widely used representations of rotation in numerical calculations [34]. In the related literature, there are mainly two different notations: Hamilton’s notation and Shuster’s notation [35]. The difference between them lies in their flipped rule for the multiplication of imaginary parts i, j, and k. Hamilton utilizes ij=k, while Shuster advocates for ij=−k to maintain the order of chain rule when transferring to direction cosine matrices (DCMs). Sommer et al. [28] surveyed this notation confusion problem in detail and argue for entirely abandoning Shuster’s notation. In this section, we present the original problem that Shuster’s notation is designed to solve and a solution for maintaining chain rule order while still using Hamilton’s notation.

A quaternion of rotation q is basically a unit quaternion; it can be defined as

(1) $$\begin{array}{c} q=\cos\frac{\theta }{2}+u^{A}\cdot \sin\frac{\theta }{2} \end{array}$$

where $u^{A}$ is the unit vector of rotation axis in frame *A*, and θ is the angle of rotation. In the rest of this article, the term “quaternion” will be used to refer to a quaternion of rotation, for the sake of simplicity.

**Figure 1:**
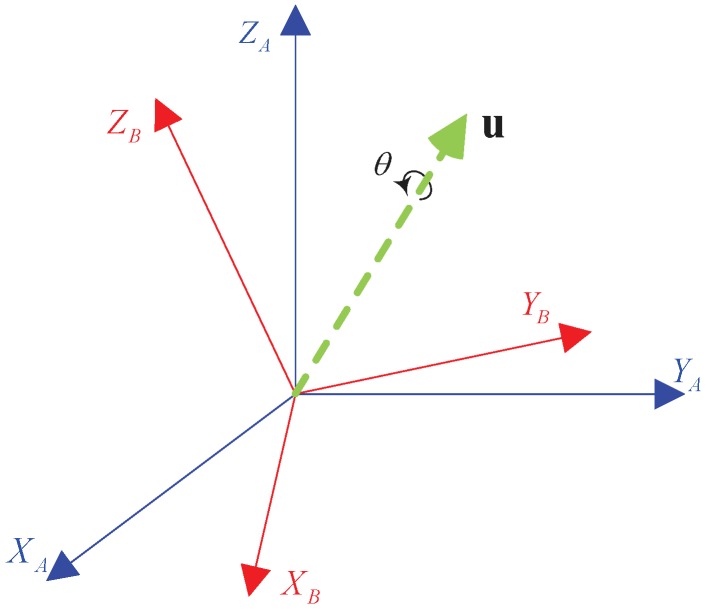
Rotation of frame *A* into frame *B*.

Equation (1) shows how to construct a quaternion q from an axis-angle $\theta u^{A}$, which describes the anticlockwise rotation of an angle θ about the axis u. If the original frame *A* is rotated to a new frame *B* after this rotation, as illustrated in Figure 1, then we can use a quaternion $q_{A}^{B}$ or a DCM $R_{A}^{B}$ to describe this rotation.

Note that $R_{A}^{B}$ can be used to compute the coordinate of a vector v in frame *B* given its coordinate in frame *A*, that is $v^{B}=R_{A}^{B}v^{A}$. $R_{A}^{B}$ can be written as a function of $q_{A}^{B}$

(2) $$\begin{array}{c} R_{A}^{B}=C_{S}\left(q_{A}^{B}\right) \end{array}$$

where $C_{S}\left(•\right)$ is an operator mapping $q_{A}^{B}$ to $R_{A}^{B}$.

Let $q_{B}^{C}$ be the quaternion describing the rotation from frame *B* to frame *C* and $q_{A}^{C}$ the rotation from frame *A* to frame *C*. Then, according to Equation (2), we have

(3) $$\begin{array}{c} \begin{array}{cc} R_{B}^{C}= & C_{S}\left(q_{B}^{C}\right)\\ R_{A}^{C}= & C_{S}\left(q_{A}^{C}\right)\;. \end{array} \end{array}$$

Coordinate transformation of vectors can also be done by applying the triple product of quaternions:

(4) $$\begin{array}{c} \begin{array}{cc} v^{B}= & {\left(q_{A}^{B}\right)}^{-1}⊗v^{A}⊗q_{A}^{B}\\ v^{C}= & {\left(q_{B}^{C}\right)}^{-1}⊗v^{B}⊗q_{B}^{C}\;. \end{array} \end{array}$$

Here we abuse the notation of $v^{A}$, $v^{B}$, and $v^{C}$ to describe quaternions with zero real part such that $v^{A}\to {\left[\begin{array}{cc} 0 & {\left(v^{A}\right)}^{T} \end{array}\right]}^{T}$. Combining the two equations above yields:

(5) $$\begin{array}{c} \begin{array}{cc} v^{C}= & {\left(q_{B}^{C}\right)}^{-1}⊗{\left(q_{A}^{B}\right)}^{-1}⊗v^{A}⊗q_{A}^{B}⊗q_{B}^{C}\\ = & {\left(q_{A}^{B}⊗q_{B}^{C}\right)}^{-1}⊗v^{A}⊗\left(q_{A}^{B}⊗q_{B}^{C}\right). \end{array} \end{array}$$

Referring to Equation (5), there is

(6) $$\begin{array}{c} q_{A}^{C}=q_{A}^{B}⊗q_{B}^{C}\;. \end{array}$$

At the same time, by applying the chain rule in DCM production, it follows that

(7) $$\begin{array}{c} R_{A}^{C}=R_{B}^{C}R_{A}^{B}\;. \end{array}$$

Now we can conclude that $C_{S}\left(q_{A}^{B}⊗q_{B}^{C}\right)=C_{S}\left(q_{B}^{C}\right)C_{S}\left(q_{A}^{B}\right)$, which means the mapping $C_{S}\left(•\right)$ is not a homomorphism. One would prefer a homomorphic mapping between DCM and quaternion to maintain the chain-rule order, which is convenient to manipulate. Shuster utilized a flipped multiplication rule to avoid this problem. This notation was adopted by the Jet Propulsion Laboratory (JPL) and thus introduced to spacecraft literatures, while other research fields were still using the traditional Hamilton notation. But as researchers have exchanged ideas between different research fields, Shuster’s notation has been utilized in robotics for rotation representation [28]. So far, all of the theories about MSCKF are deduced based on this notation [1].

As Sommer et al. [28] claimed, a homomorphic mapping could be obtained even under Hamilton’s notation. Let $C_{H}\left(•\right)$ be an operator that satisfies $C_{H}\left(q\right)=C_{S}{\left(q\right)}^{T}$. Thus, we have

(8) $$\begin{array}{c} \begin{array}{cc} R_{B}^{A}= & C_{H}\left(q_{A}^{B}\right)\\ R_{C}^{B}= & C_{H}\left(q_{B}^{C}\right)\\ R_{C}^{A}= & C_{H}\left(q_{A}^{C}\right). \end{array} \end{array}$$

According to Equations (6) and (7), we now have $C_{H}\left(q_{A}^{B}⊗q_{B}^{C}\right)=C_{H}\left(q_{A}^{B}\right)C_{H}\left(q_{B}^{C}\right)$, which proves $C_{H}\left(•\right)$ to be a homomorphism.

Given a quaternion

(9) $$\begin{array}{c} \begin{array}{cc} q_{A}^{B}= & \cos\frac{\theta }{2}+u^{A}\sin\frac{\theta }{2}\\ = & q_{0}+q_{1}i+q_{1}j+q_{2}k, \end{array} \end{array}$$

the operator $C_{H}\left(•\right)$ is defined as a function mapping quaternion $q_{A}^{B}$ to a DCM $R_{B}^{A}$ as

(10) $$\begin{array}{c} \begin{array}{cc} C_{H}\left(q_{A}^{B}\right)=\; & R_{B}^{A}\\ =\; & \left[\begin{array}{ccc} q_{0}^{2}+q_{1}^{2}-q_{2}^{2}-q_{3}^{2} & 2\left(q_{1}q_{2}-q_{0}q_{3}\right) & 2\left(q_{1}q_{3}+q_{0}q_{2}\right)\\ 2\left(q_{1}q_{2}+q_{0}q_{3}\right) & q_{0}^{2}-q_{1}^{2}+q_{2}^{2}-q_{3}^{2} & 2\left(q_{2}q_{3}-q_{0}q_{1}\right)\\ 2\left(q_{1}q_{3}-q_{0}q_{2}\right) & 2\left(q_{2}q_{3}+q_{0}q_{1}\right) & q_{0}^{2}-q_{1}^{2}-q_{2}^{2}+q_{3}^{2}. \end{array}\right] \end{array} \end{array}$$

This is, in fact, the classical Rodrigues Rotation Formula. There is a more thorough discussion about this mapping in [36].

## 3. IMU Error State Differential Equation

In this section, we deduce the IMU error state differential equation based on Hamilton’s quaternion notation. The Earth’s rotation is ignored as low cost gyros cannot measure it. The static world assumption is employed, which means that gravity has a fixed direction. This is acceptable when a VIO is working in a limited region.

### 3.1. Notation

The east-north-up geographic coordinate system at initial position is selected as the reference world frame *w*. As the Earth’s rotation is omitted, *w* can be regarded as an inertial frame. Quaternion $q_{w}^{b}$ is used to represent the rotation from frame *w* to body frame *b*. According to Equation (8), we obtain

(11) $$\begin{array}{c} C_{H}\left(q_{w}^{b}\right)=R_{b}^{w}\;. \end{array}$$

The error quaternion is defined as

(12) $$\begin{array}{c} \delta q_{w}^{b}=q_{w}^{b}⊗{\left({\hat{q}}_{w}^{b}\right)}^{-1}, \end{array}$$

where ${\hat{q}}_{w}^{b}$ is the estimated quaternion of $q_{w}^{b}$.

According to Equation (10), applying the $C_{H}\left(•\right)$ mapping to Equation (12) leads to

(13) $$\begin{array}{c} R_{w^{\prime }}^{w}=R_{b}^{w}{\left(R_{b}^{w^{\prime }}\right)}^{-1}, \end{array}$$

where $w^{\prime }$ is the estimated world frame, and $\delta q_{w}^{b}$ corresponds to the rotation between *w* and $w^{\prime }$.

$\delta q_{w}^{b}$ can be expressed in axis-angle formulation as

(14) $$\begin{array}{c} \delta q_{w}^{b}=\cos\frac{\left|\delta \theta^{w}\right|}{2}+\frac{\delta \theta^{w}}{\left|\delta \theta^{w}\right|}\sin\frac{\left|\delta \theta^{w}\right|}{2}, \end{array}$$

where $\delta \theta^{w}={\left[\begin{array}{ccc} {\delta \theta }_{x}^{w} & {\delta \theta }_{y}^{w} & {\delta \theta }_{z}^{w} \end{array}\right]}^{T}$ is an axis-angle in frame *w* that rotates frame $w^{\prime }$ to frame *w*. As $\left|\delta \theta^{w}\right|$ is a small angle, an approximate expression of Equation (14) is

(15) $$\begin{array}{c} \delta q_{w}^{b}\approx 1+\frac{1}{2}\delta \theta^{w}. \end{array}$$

Based on Equations (10) and (14), an approximate expression of $R_{w^{\prime }}^{w}$ is formulated as

(16) $$\begin{array}{c} \begin{array}{cc} R_{w^{\prime }}^{w}\approx & \left[\begin{array}{ccc} 1 & -\delta \theta_{z}^{w} & \delta \theta_{y}^{w}\\ \delta \theta_{z}^{w} & 1 & -\delta \theta_{x}^{w}\\ -\delta \theta_{y}^{w} & \delta \theta_{x}^{w} & 1 \end{array}\right]\\ = & I+\left[\delta \theta^{w}\times \right], \end{array} \end{array}$$

where the operator $\left[•\times \right]$ is used to denote the skew matrix. For a given three-dimensional (3D) vector $v={\left[\begin{array}{ccc} v_{x} & v_{y} & v_{z} \end{array}\right]}^{T}$, its skew matrix is

(17) $$\begin{array}{c} \left[v\times \right]=\left[\begin{array}{ccc} 0 & -v_{z} & v_{y}\\ v_{z} & 0 & -v_{x}\\ -v_{y} & v_{x} & 0 \end{array}\right]. \end{array}$$

### 3.2. IMU Measurement Model

An IMU includes a 3-axis gyroscope and a 3-axis accelerometer, whose axes are aligned with the body frame. The output of the gyroscope is modeled as

(18) $$\begin{array}{c} \omega_{m}^{b}=\omega_{wb}^{b}+b_{g}+n_{g}, \end{array}$$

where $\omega_{wb}^{b}$ is the true angular velocity, $b_{g}$ denotes the gyroscope bias under the body frame, and $n_{g}$ is the Gaussian white noise.

The accelerometer measures the specific force along a body-fixed axis, which includes an opposite gravity and is affected by bias and noise as well:

(19) $$\begin{array}{c} \begin{array}{cc} f_{m}^{b}=\; & a^{b}-R_{w}^{b}g^{w}+b_{a}+n_{a}\\ =\; & R_{w}^{b}\left(a^{w}-g^{w}\right)+b_{a}+n_{a}, \end{array} \end{array}$$

where $a^{b}$ is the true acceleration, and $g^{w}={\left[\begin{array}{ccc} 0 & 0 & -g \end{array}\right]}^{T}$ denotes the gravity under the world frame. $b_{a}$ and $n_{a}$ denote the bias and the Gaussian white noise under the body frame, respectively.

The biases $b_{g}$ and $b_{a}$ are modeled as random walk processes

(20) $$\begin{array}{c} \begin{array}{cc} {\dot{b}}_{g}= & n_{wg}\\ {\dot{b}}_{a}= & n_{wa}, \end{array} \end{array}$$

where $n_{wg}$ and $n_{wa}$ are Gaussian white noises.

### 3.3. IMU Error State Definition

The IMU state includes the quaternion $q_{w}^{b}$, velocity $v_{b}^{w}$ and position $p_{b}^{w}$ of the body frame origin in the world frame, and IMU biases $b_{g}$ and $b_{a}$. The IMU state can be defined as

(21) $$\begin{array}{c} x_{IMU}={\left[\begin{array}{ccccc} {q_{w}^{b}}^{T} & {v_{b}^{w}}^{T} & {p_{b}^{w}}^{T} & b_{g}^{T} & b_{a}^{T} \end{array}\right]}^{T}. \end{array}$$

The filter is designed based on the error state because it is convenient to process by extended Kalman filter (EKF). Three dimensional angular error $\delta \theta^{w}$ rather than four dimensional quaternion error $\delta q_{w}^{b}$ is utilized since it is accordance with the degree of freedom (DOF) of rotation, and thus a minimum parameterization.

Other error state components are simply defined as the Euclidean distances between true states and the estimated states, which lead to

(22) $$\begin{array}{cc} \delta v_{b}^{w}= & v_{b}^{w}-{\hat{v}}_{b}^{w}, \end{array}$$

(23) $$\begin{array}{cc} \delta p_{b}^{w}= & p_{b}^{w}-{\hat{p}}_{b}^{w}, \end{array}$$

(24) $$\begin{array}{cc} \delta b_{g}= & b_{g}-{\hat{b}}_{g}, \end{array}$$

(25) $$\begin{array}{cc} \delta b_{a}= & b_{a}-{\hat{b}}_{a}. \end{array}$$

The overall IMU error state can now be concluded as

(26) $$\begin{array}{c} \delta x_{IMU}={\left[\begin{array}{ccccc} \delta {\theta^{w}}^{T} & \delta {v_{b}^{w}}^{T} & \delta {p_{b}^{w}}^{T} & b_{g}^{T} & b_{a}^{T} \end{array}\right]}^{T}. \end{array}$$

### 3.4. Differential Equation

The matrix form of the differential equation of the overall IMU error state is as follows.

(27) $$\begin{array}{c} \delta {\dot{x}}_{IMU}=F\delta x_{IMU}+Gn_{IMU}, \end{array}$$

where $n_{IMU}$ denotes the IMU noise, given by

(28) $$\begin{array}{cc} \delta x_{IMU}= & {\left[\begin{array}{ccccc} \delta \theta^{w} & \delta {v_{b}^{w}}^{T} & \delta {p_{b}^{w}}^{T} & \delta b_{g}^{T} & \delta b_{a}^{T} \end{array}\right]}^{T}, \end{array}$$

(29) $$\begin{array}{cc} n_{IMU}= & {\left[\begin{array}{cccc} n_{g}^{T} & n_{a}^{T} & n_{wg}^{T} & n_{wa}^{T} \end{array}\right]}^{T}, \end{array}$$

and the matrices F and G are as follows:

(30) $$\begin{array}{cc} F= & \left[\begin{array}{ccccc} 0_{3\times 3} & 0_{3\times 3} & 0_{3\times 3} & -{\hat{R}}_{b}^{w} & 0_{3\times 3}\\ -\left[\left({\hat{R}}_{b}^{w}\hat{a}\right)\times \right] & 0_{3\times 3} & 0_{3\times 3} & 0_{3\times 3} & -{\hat{R}}_{b}^{w}\\ 0_{3\times 3} & I_{3\times 3} & 0_{3\times 3} & 0_{3\times 3} & 0_{3\times 3}\\ 0_{3\times 3} & 0_{3\times 3} & 0_{3\times 3} & 0_{3\times 3} & 0_{3\times 3}\\ 0_{3\times 3} & 0_{3\times 3} & 0_{3\times 3} & 0_{3\times 3} & 0_{3\times 3} \end{array}\right], \end{array}$$

(31) $$\begin{array}{cc} G= & \left[\begin{array}{cccc} -{\hat{R}}_{b}^{w} & 0_{3\times 3} & 0_{3\times 3} & 0_{3\times 3}\\ 0_{3\times 3} & -{\hat{R}}_{b}^{w} & 0_{3\times 3} & 0_{3\times 3}\\ 0_{3\times 3} & 0_{3\times 3} & 0_{3\times 3} & 0_{3\times 3}\\ 0_{3\times 3} & 0_{3\times 3} & I_{3\times 3} & 0_{3\times 3}\\ 0_{3\times 3} & 0_{3\times 3} & 0_{3\times 3} & I_{3\times 3} \end{array}\right]. \end{array}$$

## 4. Fully Linear State Transition Equation Formulation

A state transition equation is needed for the extended Kalman filter (EKF) to propagate the state and covariance. One commonly used method is to make a first-order approximation based on a continuous differential equation [37]. Li and Mourikis [19] proposed a closed-form error state transition equation that effectuated a system model with no information loss. However, there are still some tricky integration terms left behind. In this section, we first present the closed-form IMU error state transition equation based on the results of Section 3. Then, we solve the integration terms by two-sample fitting of the rotation matrix, resulting in a closed-form formulation that is fully linear.

### 4.1. Original Closed-Form Equation

Following the methodology of Li and Mourikis [19], the closed-form transition equation was deduced and presented in what follows. Noting that *k* and k+1 are consecutive discrete sampling instants of IMU, and Δt is the sampling period,

(32) $$\begin{array}{cc} \delta \theta_{k+1|k}^{w}= & \Phi_{\theta \theta }\left(k+1,k\right)\delta \theta_{k|k}^{w}+\Phi_{\theta b_{g}}\left(k+1,k\right)\delta b_{gk|k}+n_{\theta k}, \end{array}$$

(33) $$\begin{array}{c} \begin{array}{cc} \delta v_{k+1|k}^{w}= & \Phi_{v\theta }\left(k+1,k\right)\delta \theta_{k|k}^{w}+\Phi_{vv}\left(k+1,k\right)\delta v_{k|k}^{w}\\ & +\Phi_{vb_{g}}\left(k+1,k\right)\delta b_{gk|k}+\Phi_{vb_{a}}\left(k+1,k\right)\delta b_{ak|k}+n_{vk}, \end{array} \end{array}$$

(34) $$\begin{array}{c} \begin{array}{cc} \delta p_{k+1|k}^{w}= & \Phi_{p\theta }\left(k+1,k\right)\delta \theta_{k|k}^{w}+\Phi_{pv}\left(k+1,k\right)\delta v_{k|k}^{w}+\Phi_{pp}\left(k+1,k\right)\delta p_{k|k}^{w}\\ & +\Phi_{pb_{g}}\left(k+1,k\right)\delta b_{gk|k}+\Phi_{pb_{a}}\left(k+1,k\right)\delta b_{ak|k}+n_{pk}, \end{array} \end{array}$$

(35) $$\begin{array}{cc} \delta b_{gk+1|k}= & \Phi_{b_{g}b_{g}}\left(k+1,k\right)\delta b_{gk|k}+n_{b_{g}k}, \end{array}$$

(36) $$\begin{array}{cc} \delta b_{ak+1|k}= & \Phi_{b_{a}b_{a}}\left(k+1,k\right)\delta b_{ak|k}+n_{b_{a}k}, \end{array}$$

where $\Phi_{x_{1}x_{2}}$ is used to represent the transition matrix of the error state of $x_{1}$ with respect to the error state of $x_{2}$, and $n_{*}$ terms represent noise. All of the $\Phi_{*}$ and $n_{*}$ terms are listed as follows:

(37) $$\begin{array}{cc} \Phi_{\theta \theta }\left(k+1,k\right)= & I_{3\times 3}, \end{array}$$

(38) $$\begin{array}{cc} \Phi_{\theta b_{g}}\left(k+1,k\right)= & -{\hat{R}}_{b_{k}}^{w}\int_{t_{k}}^{t_{k+1}}{\hat{R}}_{b_{\tau }}^{b_{k}}d\tau , \end{array}$$

(39) $$\begin{array}{cc} \Phi_{v\theta }\left(k+1,k\right)= & -\left[\left({\hat{v}}_{k+1|k}^{w}-{\hat{v}}_{k|k}^{w}-g^{w}\Delta t\right)\times \right], \end{array}$$

(40) $$\begin{array}{cc} \Phi_{vv}\left(k+1,k\right)= & I_{3\times 3}, \end{array}$$

(41) $$\begin{array}{cc} \Phi_{vb_{g}}\left(k+1,k\right)= & \int_{t_{k}}^{t_{k+1}}\left\{\left[\left({\dot{\hat{v}}}_{\tau }^{w}-g^{w}\right)\times \right]{\hat{R}}_{b_{k}}^{w}\left(\int_{t_{k}}^{\tau }{\hat{R}}_{b_{m}}^{b_{k}}dm\right)\right\}d\tau , \end{array}$$

(42) $$\begin{array}{cc} \Phi_{vb_{a}}\left(k+1,k\right)= & -{\hat{R}}_{b_{k}}^{w}\int_{t_{k}}^{t_{k+1}}{\hat{R}}_{b_{\tau }}^{b_{k}}d\tau , \end{array}$$

(43) $$\begin{array}{cc} \Phi_{p\theta }\left(k+1,k\right)= & -\left[\left({\hat{p}}_{k+1|k}^{w}-{\hat{p}}_{k|k}^{w}-{\hat{v}}_{k|k}^{w}\Delta t-\frac{1}{2}g^{w}\Delta t^{2}\right)\times \right], \end{array}$$

(44) $$\begin{array}{cc} \Phi_{pv}\left(k+1,k\right)= & I_{3\times 3}\Delta t, \end{array}$$

(45) $$\begin{array}{cc} \Phi_{pp}\left(k+1,k\right)= & I_{3\times 3}, \end{array}$$

(46) $$\begin{array}{cc} \Phi_{pb_{g}}\left(k+1,k\right)= & \int_{t_{k}}^{t_{k+1}}\left\{\int_{t_{k}}^{\tau }\left(\left[\left({\dot{\hat{v}}}_{s}^{w}-g^{w}\right)\times \right]{\hat{R}}_{b_{k}}^{w}\left[\int_{t_{k}}^{s}{\hat{R}}_{b_{m}}^{b_{k}}dm\right]\right)ds\right\}d\tau , \end{array}$$

(47) $$\begin{array}{cc} \Phi_{pb_{a}}\left(k+1,k\right)= & -{\hat{R}}_{b_{k}}^{w}\int_{t_{k}}^{t_{k+1}}\left(\int_{t_{k}}^{\tau }{\hat{R}}_{b_{s}}^{b_{k}}ds\right)d\tau , \end{array}$$

(48) $$\begin{array}{cc} \Phi_{b_{g}b_{g}}\left(k+1,k\right)= & I_{3\times 3}, \end{array}$$

(49) $$\begin{array}{cc} \Phi_{b_{a}b_{a}}\left(k+1,k\right)= & I_{3\times 3}, \end{array}$$

(50) $$\begin{array}{cc} n_{\theta k}= & {\hat{R}}_{b_{k}}^{w}\int_{t_{k}}^{t_{k+1}}{\hat{R}}_{b_{\tau }}^{b_{k}}\left(\int_{t_{k}}^{\tau }n_{wgs}ds+n_{g\tau }\right)d\tau , \end{array}$$

(51) $$\begin{array}{c} \begin{array}{cc} n_{vk}= & \int_{t_{k}}^{t_{k+1}}{\hat{R}}_{b_{\tau }}^{w}\left(-\int_{t_{k}}^{\tau }n_{was}ds-n_{a\tau }\right)d\tau \\ & -\int_{t_{k}}^{t_{k+1}}\left\{\left[\left({\dot{\hat{v}}}_{\tau }^{w}-g^{w}\right)\times \right]{\hat{R}}_{b_{k}}^{w}\left[\int_{t_{k}}^{\tau }{\hat{R}}_{b_{m}}^{b_{k}}\left(\int_{t_{k}}^{m}n_{wgs}ds+n_{gm}\right)dm\right]\right\}d\tau , \end{array} \end{array}$$

(52) $$\begin{array}{cc} n_{pk}= & \int_{t_{k}}^{t_{k+1}}n_{v\tau }d\tau , \end{array}$$

(53) $$\begin{array}{cc} n_{b_{g}k}= & \int_{t_{k}}^{t_{k+1}}n_{wg\tau }d\tau , \end{array}$$

(54) $$\begin{array}{cc} n_{b_{a}k}= & \int_{t_{k}}^{t_{k+1}}n_{wa\tau }d\tau . \end{array}$$

### 4.2. Fully Linear Closed-Form Formulation

Notice that in Equations (38), (41), (42), (46), and (47), although they are closed-form expressions, there are still some tricky integration terms that are not straightforward for implementation. One can solve these terms with numerical integration, but here we present a fully linear analytical expression that is readily implemented. The key is to solve $\int_{t_{k}}^{t_{k+1}}{\hat{R}}_{b_{\tau }}^{b_{k}}d\tau$. We first apply a two-sample fitting method to approximate the axis-angle representing ${\hat{R}}_{b_{\tau }}^{b_{k}}$, then Rodrigues’ rotation formula is applied to express the DCM as a linear function of τ, thus making the integration easy to solve.

#### 4.2.1. Two-Sample Fitting of Axis-Angle

Any DCM can be regarded as a single rotation about a fixed axis, and thus can be represented by an axis-angle. Let the axis-angle of ${\hat{R}}_{b_{k+1}}^{b_{k}}$ be $\phi =\alpha u^{b_{k}}$, where α is the angle of rotation and $u^{b_{k}}$ is the rotation axis. Let τ be a time instant between $t_{k}$ and $t_{k+1}$ and $\varepsilon =\tau -t_{k}$, then a linear model can be used to represent the axis-angle of ${\hat{R}}_{b_{\tau }}^{b_{k}}$:

(55) $$\begin{array}{c} \begin{array}{c} \phi_{\tau }=\phi \left(\varepsilon \right)=\frac{\varepsilon }{\Delta t}\alpha u^{b_{k}}=\frac{\varepsilon }{\Delta t}\phi . \end{array} \end{array}$$

Angular velocity measurements of $t_{k}$ and $t_{k+1}$ are available when calculating the transition matrix $\Phi \left(k+1,k\right)$, so a two-sample fitting method can be used to approximate the axis-angle ϕ. We start from the differential equation of $\phi \left(\varepsilon \right)$ [36]:

(56) $$\begin{array}{c} \dot{\phi }\left(\varepsilon \right)=\bar{\omega }+\frac{1}{2}\phi \left(\varepsilon \right)\times \bar{\omega }+\frac{1}{12}\phi \left(\varepsilon \right)\times \left(\phi \left(\varepsilon \right)\times \bar{\omega }\right), \end{array}$$

where $\bar{\omega }$ is the average angular velocity between $t_{k}$ and $t_{k+1}$. As two gyro measurements are available, we use a straight line model to fit $\bar{\omega }$ as

(57) $$\begin{array}{c} \bar{\omega }\left(t_{k}+\varepsilon \right)=a+2b\varepsilon ,\;0\leq \varepsilon \leq \Delta t. \end{array}$$

Considering ${\bar{\omega }}^{b}\left(t_{k}\right)={\hat{\omega }}_{wb}^{b}\left(t_{k}\right)$ and ${\bar{\omega }}^{b}\left(t_{k}+\Delta t\right)={\hat{\omega }}_{wb}^{b}\left(t_{k+1}\right)$, leads to

(58) a=ω^wbbtkb=ω^wbbtk+1−ω^wbbtkω^wbbk+1−ω^wbbk2Δt2Δt.

According to Equation (55), ϕ is equal to $\phi_{t_{k+1}}$, then using Taylor expansion to expand ϕ at linearized point $t_{k}$ yields

(59) $$\begin{array}{c} \begin{array}{cc} \phi = & \phi_{t_{k+1}}\\ = & \phi_{t_{k}}+\Delta t{\dot{\phi }}_{t_{k}}+\frac{\Delta t^{2}}{2!}{\ddot{\phi }}_{t_{k}}+\cdots \\ = & \phi \left(0\right)+\Delta t\dot{\phi }\left(0\right)+\frac{\Delta t^{2}}{2!}\ddot{\phi }\left(0\right)+\cdots . \end{array} \end{array}$$

Now define a new function of ε as

(60) $$\begin{array}{c} \Delta \theta \left(\varepsilon \right)=\int_{0}^{\varepsilon }{\hat{\omega }}_{wb}^{b}\left(t_{k}+\varepsilon \right)d\varepsilon . \end{array}$$

It can be pointed out that $\phi \left(\varepsilon \right)\approx \Delta \theta \left(\varepsilon \right)$. Derivatives of $\Delta \theta \left(0\right)$ are defined as

(61) $$\begin{array}{c} \begin{array}{cc} \Delta \theta \left(0\right)= & 0,\\ \Delta \dot{\theta }\left(0\right)= & {\hat{\omega }}_{wb}^{b}\left(t_{k}\right)=a,\\ \Delta \ddot{\theta }\left(0\right)= & {\dot{\hat{\omega }}}_{wb}^{b}\left(t_{k}\right)=2b,\\ \Delta \theta^{\left(i\right)}\left(0\right)= & \hat{\omega }{{}_{wb}^{b}}^{\left(i-1\right)}\left(t_{k}\right)=0,\;i=3,4,5,\cdots . \end{array} \end{array}$$

The third term in Equation (56) is a high-order small quantity that can be omitted. By substituting $\phi \left(\varepsilon \right)$ as $\Delta \theta \left(\varepsilon \right)$, Equation (56) turns into

(62) $$\begin{array}{c} \dot{\phi }\left(\varepsilon \right)=\bar{\omega }\left(t_{k}+\varepsilon \right)+\frac{1}{2}\Delta \theta \left(\varepsilon \right)\times \bar{\omega }\left(t_{k}+\varepsilon \right). \end{array}$$

Now the high-order derivatives of $\phi \left(\varepsilon \right)$ can be obtained:

(63) $$\begin{array}{c} \begin{array}{cc} \ddot{\phi }\left(\varepsilon \right)= & \dot{\bar{\omega }}\left(t_{k}+\varepsilon \right)+\frac{1}{2}\Delta \dot{\theta }\left(\varepsilon \right)\times \bar{\omega }\left(t_{k}+\varepsilon \right)+\frac{1}{2}\Delta \theta \left(\varepsilon \right)\times \dot{\bar{\omega }}\left(t_{k}+\varepsilon \right),\\ \phi^{\left(3\right)}\left(\varepsilon \right)= & \frac{1}{2}\Delta \ddot{\theta }\left(\varepsilon \right)\times \bar{\omega }\left(t_{k}+\varepsilon \right)+\Delta \dot{\theta }\left(\varepsilon \right)\times \dot{\bar{\omega }}\left(t_{k}+\varepsilon \right),\\ \phi^{\left(4\right)}\left(\varepsilon \right)= & \frac{3}{2}\Delta \ddot{\theta }\left(\varepsilon \right)\times \dot{\bar{\omega }}\left(t_{k}+\varepsilon \right),\\ \phi^{\left(i\right)}\left(\varepsilon \right)= & 0,\;i=5,6,7,\cdots . \end{array} \end{array}$$

Let ε=0, and considering Equation (61), we have

(64) $$\begin{array}{c} \begin{array}{cc} \phi \left(0\right)= & 0,\\ \dot{\phi }\left(0\right)= & a,\\ \ddot{\phi }\left(0\right)= & 2b,\\ \phi^{\left(3\right)}\left(0\right)= & a\times b,\\ \phi^{\left(i\right)}\left(0\right)= & 0,i=4,5,6,\cdots . \end{array} \end{array}$$

Substituting the equations above into Equation (59) yields

(65) $$\begin{array}{c} \begin{array}{cc} \phi = & a\Delta t+b\Delta t^{2}+\frac{1}{6}\left(a\times b\right)\Delta t^{3}\\ = & \frac{1}{2}\left({\hat{\omega }}_{wb}^{b}\left(k\right)+{\hat{\omega }}_{wb}^{b}\left(k+1\right)\right)\Delta t+\frac{1}{12}\left({\hat{\omega }}_{wb}^{b}\left(k\right)\times {\hat{\omega }}_{wb}^{b}\left(k+1\right)\right)\Delta t^{2}. \end{array} \end{array}$$

This is how the axis-angle between two consecutive sampling time instants $t_{k}$ and $t_{k+1}$ can be computed.

According to Rodrigues’ rotation formula,

(66) $$\begin{array}{c} {\hat{R}}_{b_{k+1}}^{b_{k}}=I+\sin\alpha \left[u^{b_{k}}\times \right]+\left(1-\cos\alpha \right){\left[u^{b_{k}}\times \right]}^{2}. \end{array}$$

As α is a small angular, since Δt is small, Equation (66) has an approximation

(67) $$\begin{array}{c} \begin{array}{cc} {\hat{R}}_{b_{k+1}}^{b_{k}}\approx & I+\alpha \left[u^{b_{k}}\times \right]\\ = & I+\left[\phi \times \right]. \end{array} \end{array}$$

Now, substituting Equation (55) into Equation (67) leads to

(68) $$\begin{array}{c} {\hat{R}}_{b_{\tau }}^{b_{k}}\approx I+\frac{\tau -t_{k}}{\Delta t}\left[\phi \times \right]. \end{array}$$

Finally, the general procedure to solve the integration term $\int_{t_{k}}^{t_{k+1}}{\hat{R}}_{b_{\tau }}^{b_{k}}d\tau$ can be summarized as follows:

1. Compute the axis-angle between $t_{k}$ and $t_{k+1}$ according to Equation (65).
2. Express ${\hat{R}}_{b_{\tau }}^{b_{k}}$ as Equation (68).
3. Easily solve the $\int_{t_{k}}^{t_{k+1}}{\hat{R}}_{b_{\tau }}^{b_{k}}d\tau$ term, as it becomes an integration about a linear analytic expression.

#### 4.2.2. Solve Integration Terms in $\Phi_{*}$

The fully linear closed-form transition matrix of Equations (38), (41), (42), (46), and (47) can now be obtained by simply solving the integration terms. The results are listed below.

(69) $$\begin{array}{c} \begin{array}{cc} \Phi_{\theta b_{g}}\left(k+1,k\right)= & -{\hat{R}}_{b_{k}}^{w}\left(\Delta tI+\frac{1}{2}\Delta t\left[\phi \times \right]\right),\\ \Phi_{vb_{g}}\left(k+1,k\right)= & \left[\left(-{\hat{p}}_{k+1|k}^{w}+{\hat{p}}_{k|k}^{w}+{\hat{v}}_{k+1|k}^{w}\Delta t-\frac{1}{2}g^{w}\Delta t^{2}\right)\times \right]{\hat{R}}_{b_{k}}^{w}\\ & +\left[\left(-\frac{1}{2}{\hat{p}}_{k+1|k}^{w}+\frac{1}{2}{\hat{p}}_{k|k}^{w}+\frac{1}{2}{\hat{v}}_{k+1|k}^{w}\Delta t-\frac{1}{6}g^{w}\Delta t^{2}\right)\times \right]{\hat{R}}_{b_{k}}^{w}\left[\phi \times \right],\\ \Phi_{vb_{a}}\left(k+1,k\right)= & -{\hat{R}}_{b_{k}}^{w}\left(\Delta tI+\frac{1}{2}\Delta t\left[\phi \times \right]\right),\\ \Phi_{pb_{g}}\left(k+1,k\right)= & \left[\left(-\frac{1}{6}g^{w}\Delta t^{3}\right)\times \right]{\hat{R}}_{b_{k}}^{w}\\ & +\left[\left(\frac{1}{4}{\hat{p}}_{k+1|k}^{w}\Delta t-\frac{1}{4}{\hat{p}}_{k|k}^{w}\Delta t-\frac{1}{24}g^{w}\Delta t^{3}\right)\times \right]{\hat{R}}_{b_{k}}^{w}\left[\phi \times \right],\\ \Phi_{pb_{a}}\left(k+1,k\right)= & -\frac{1}{6}{\hat{R}}_{b_{k}}^{w}\Delta t^{2}\left(3I+\left[\phi \times \right]\right). \end{array} \end{array}$$

Notice that all of the variables needed are available at the time of calculating the Φ terms above. This model is unbiased up to the information loss of the two-sample fitting of DCM, which is small due to the utilization of all related measurement data.

#### 4.2.3. Process Noise Terms

The property of noise terms in Equations (50)–(54) should be acquired to compute the process noise covariance matrix in a Kalman filter. The process noise covariance at $t_{k}$ can be computed as [37]:

(70) $$\begin{array}{c} Q\left(t_{k}\right)=\int_{t_{k}}^{t_{k+1}}\Phi \left(t_{k+1},\tau \right)G\left(\tau \right)qG^{T}\left(\tau \right)\Phi^{T}\left(t_{k+1},\tau \right)d\tau . \end{array}$$

We temporarily abuse symbol q here to represent the noise intensity matrix. Φ is the overal; IMU error state transition matrix. As Δt is a small quantity, an approximate expression of Equation (70) is formulated as

(71) $$\begin{array}{c} Q\left(t_{k}\right)\approx \Phi \left(t_{k+1},t_{k}\right)G\left(t_{k}\right)qG^{T}\left(t_{k}\right)\Phi^{T}\left(t_{k+1},t_{k}\right)\Delta t. \end{array}$$

The discrete form, which will be preferable for a discrete filter implementation, is

(72) $$\begin{array}{c} Q\left(k\right)\approx \Phi \left(k+1,k\right)G\left(k\right)qG^{T}\left(k\right)\Phi^{T}\left(k+1,k\right)\Delta t. \end{array}$$

### 4.3. Summarization

According to the derivation above, the proposed fully linear closed-form IMU error state transition equation is as follows:

(73) $$\begin{array}{c} \delta x_{IMU}\left(k+1\right)=\Phi \left(k+1,k\right)\delta x_{IMU}\left(k\right)+n_{IMU}\left(k\right), \end{array}$$

where

(74) $$\begin{array}{cc} \Phi \left(k+1,k\right)= & \left[\begin{array}{ccccc} I_{3\times 3} & 0_{3\times 3} & 0_{3\times 3} & \Phi_{\theta b_{g}} & 0_{3\times 3}\\ \Phi_{v\theta } & I_{3\times 3} & 0_{3\times 3} & \Phi_{vb_{g}} & \Phi_{vb_{a}}\\ \Phi_{p\theta } & \Delta tI_{3\times 3} & I_{3\times 3} & \Phi_{pb_{g}} & \Phi_{pb_{a}}\\ 0_{3\times 3} & 0_{3\times 3} & 0_{3\times 3} & I_{3\times 3} & 0_{3\times 3}\\ 0_{3\times 3} & 0_{3\times 3} & 0_{3\times 3} & 0_{3\times 3} & I_{3\times 3} \end{array}\right], \end{array}$$

(75) $$\begin{array}{cc} n_{IMU}\left(k\right)= & {\left[\begin{array}{ccccc} n_{\theta k}^{T} & n_{vk}^{T} & n_{pk}^{T} & n_{b_{g}k}^{T} & n_{b_{a}k}^{T} \end{array}\right]}^{T}, \end{array}$$

and the covariance matrix of $n_{IMU}\left(k\right)$ is $E\left[n_{IMU}\left(k\right)n_{IMU}^{T}\left(k\right)\right]=Q\left(k\right)$.

The integration terms are solved using a fitting rule of DCMs by utilizing all of the related measurements, so we claim that the obtained formulation is an unbiased model up to the numerical integration resolution.

## 5. ORB Descriptor-Assisted Optical Flow Front-End

In this section, we propose a sparse visual front-end using descriptor-assisted optical flow feature tracking.

Different kinds of feature descriptors are used in several VIOs to accomplish feature extraction and matching [1,6,30]. In contrast, other solutions choose optical flow feature tracking as their front-end solution since it is not that time-consuming compared to the descriptor-based methods [2,23,24]. However, there are more wrong matches in optical flow tracking than in descriptor-based methods, and these wrong matches exist even after eliminating algorithms such as random sample consensus (RANSAC). Filter-based VIOs are very sensitive to feature outliers since they don’t eliminate outliers in their iterations as the optimization-based ones do. Wrong matches left behind will participate in measurement updates, which may result in deteriorating estimates or even failure. As a conclusion, a robust front-end is needed to achieve stable performance for filter-based VIOs, while a real-time solution also calls for fast data correlation.

Yang et al. [29], refined ORB-SLAM [38] by using a sparse optical flow algorithm. The key idea was to correct the image coordinates of ORB features by optical flow tracking results to achieve sub-pixel precision. The proposed method here is a bit different since we use optical flow to first conduct a fast tracking, then compute descriptor distance between matched feature pair members and justify whether they are a good match-up.

There exist plenty of feature descriptor algorithms. We chose the ORB descriptor in our proposed method for two reasons:

1. The ORB descriptor is a binary string, so the distance between two descriptors can be expressed as a Hamming distance, which can be computed efficiently.
2. The rotation between consecutive images in a real-time application is usually very gentle, so invariance to rotation is not very important for a descriptor.

### Descriptor Distance Analysis for General Corner Features

The basic visual front-end is based on Shi-Tomasi corner detection [39] and optical flow tracking [40]. It is important to figure out whether the ORB descriptor is meaningful for general Shi-Tomasi corner features. An experiment was done and proved that it is indeed meaningful statistically. We calculated the feature angle for a Shi-Tomasi feature and then used it to compute the ORB descriptor [32]. Several tests were conducted in the experiment. For each test, feature pairs from every two adjacent images of a continuous image stream were stored separately in two sequences. These tests basically analyzed the statistical properties of ORB descriptor distances of feature pairs, including

1. Coarsely matched feature pairs based on Shi-Tomasi corner detection and optical flow tracking.
2. Relatively strictly matched feature pairs based on ORB descriptor matching and RANSAC.
3. Randomly constructed feature pairs.
4. Unmatched feature pairs generated by inverse order of one of the strictly matched feature sequences.

**Figure 2:**
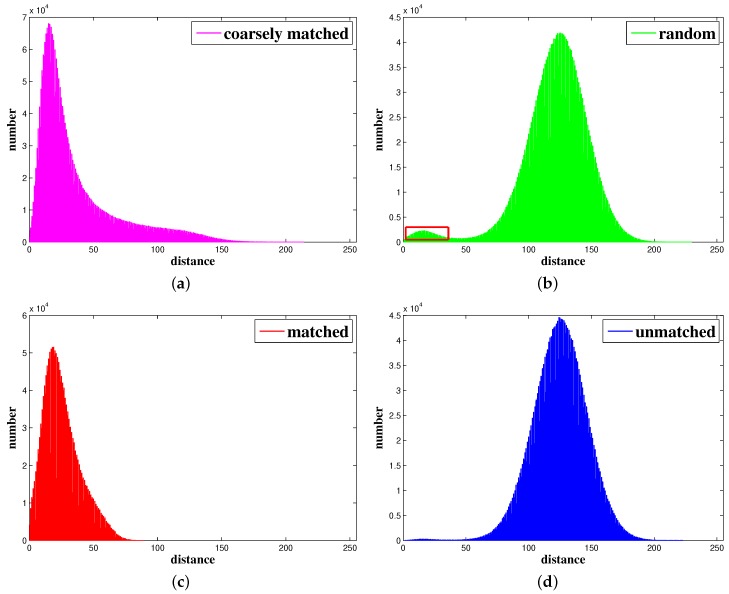
Statistical distribution of ORB descriptor [32] distances for coarsely matched, strictly matched, random constructed, and unmatched Shi-Tomasi feature pairs. The X axis represents descriptor distances and ranges from 0 to 255. The range of the Y axis is determined by the number of feature pairs in each experiment. (**a**) Coarsely matched features results. (**b**) Random constructed features results. (**c**) Strictly matched features results. (**d**) Unmatched features results.

One feature sequence from strictly matched pairs was inverted to generate strictly unmatched feature pairs. The experimental result is shown in Figure 2.

We strongly suspect the very long tail in Figure 2a may be due to wrong matches because no further outlier rejection method was applied after optical flow tracking in this test. In Figure 2b, except for the massive Guassian-like distribution, a little bump centered at about 17 appeared, which is framed by a red rectangular border. This is because the random pairs were constructed in two adjacent images and thus, two matched features have a considerable probability of being coincidentally formed into a pair. These two experiments prove that ORB descriptors and descriptor distances are meaningful for general Shi-Tomasi corners, from a statistical standpoint.

**Figure 3:**
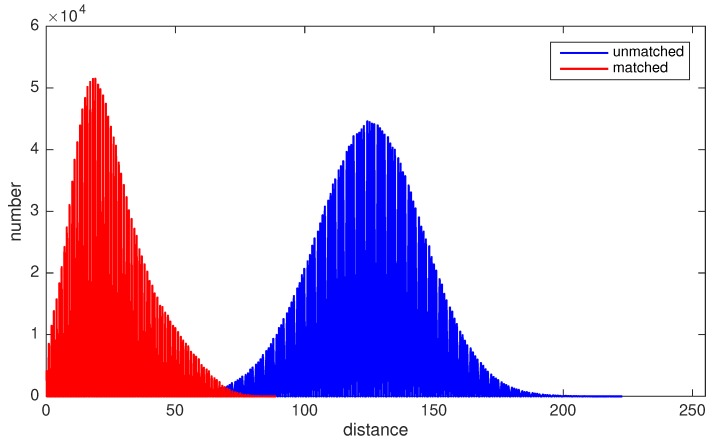
This figure shows the descriptor distances of unmatched and matched feature pairs. It can be clearly seen that the difference is statistically significant, thus a heuristic algorithm can be used to pick out outliers.

In order to clearly analyze the statistical properties of matched and unmatched pairs, two further tests were conducted. First, a descriptor-based matching and RANSAC mechanism were applied to obtain relatively strictly matched feature pairs. Then, the order of one of the feature sequences was reversed, which is a simple yet effective way to make two sequences unmatched. Descriptor distances before and after order reversion were computed, and statistical results are shown in Figure 2c,d. It can be seen from the figures that the long tail and little bump disappear because of the relatively strict pairing rule. They are plotted together in Figure 3 to make a clear comparison.

The experimental results show that the descriptor distances of unmatched and matched feature pairs possess significantly different statistical properties. As shown in Figure 3, descriptor distances of unmatched features approximately follow the Gaussian distribution with a mean, or we can say peak, at about 124.7 and with a standard deviation of 21.8. For matched pairs, the distribution shows a sharper peak at about 18.5. There is still a tail in the matched distribution, but it is much smaller than the one in Figure 2a. The difference between matched and unmatched pairs is significant enough to design a strategy to filter out wrong matches.

We use a heuristic to complete the mission:

- For feature pairs with distances lower than the smaller peak value, classify them as inliers.
- For feature pairs with distances higher than the bigger peak value, classify them as outliers.
- For feature pairs whose distances are between two peaks, calculate and compare the Mahalanobis distances to both peak to decide their classification.

## 6. EKF-Based VIO Implementation Details and Improvements

**Figure 4:**
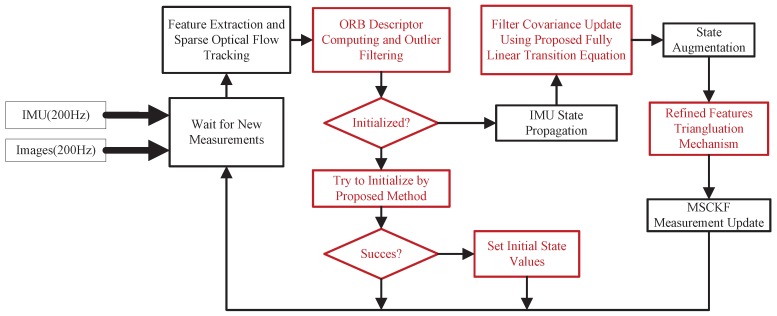
Flow chart of extended Kalman filter (EKF)-based visual-inertial odometry (VIO) implementation. Red sections highlight novelties proposed in this paper. Term “IMU” stands for inertial measurement unit, and term “MSCKF” stands for multi-state constraint Kalman filter.

In this section, implementation details and improvements of the proposed EKF-based VIO are presented, including filtering scheme, automatic initialization procedure, and refined feature triangulation mechanism. An overall flow chart of the implemented VIO algorithm is shown in Figure 4. Red sections highlight novelties proposed in this paper.

### 6.1. Filter State and Measurement Model

A VIO following the scheme of Mourikis and Roumeliotis [1] is implemented. The system state includes a sliding window of *N* historical IMU poses and camera-IMU extrinsic as proposed by Li and Mourikis [22]. The overall system state is formulated as

(76) $$\begin{array}{c} x_{all}={\left[\begin{array}{cccccccc} x_{IMU} & q_{b}^{c} & p_{c}^{b} & q_{w}^{b_{1}} & p_{b_{1}}^{w} & \cdots & q_{w}^{b_{N}} & p_{b_{N}}^{w} \end{array}\right]}^{T}. \end{array}$$

Therefore, the overall error state of the filter is

(77) $$\begin{array}{c} \delta x_{all}={\left[\begin{array}{cccccccc} \delta x_{IMU} & \delta \theta^{b} & \delta p_{c}^{b} & \delta \theta_{1}^{w} & \delta p_{b_{1}}^{w} & \cdots & \delta \theta_{N}^{w} & \delta p_{b_{N}}^{w} \end{array}\right]}^{T}. \end{array}$$

The measurement residual is a linearized residual about historical IMU pose errors and camera-IMU extrinsic errors. The original reprojection error is manipulated firstly by left nullspace multiplication to marginalize out the feature position, and secondly by applying QR decomposition to decrease the residual dimensions without information loss [1]. Furthermore, only residuals passing through the Mahalanobis gating test would be used in measurement updating.

### 6.2. Automatic Initialization Procedure

An automatic initialization procedure is developed. Firstly, a stationary scene is automatically detected by only using image stream. Secondly, stationary IMU data is used to initialize the system state. The detailed procedure is described in Algorithm 1.

The algorithm identifies a stationary scene by continuously detecting almost no motion of tracked features. Then, static gyro data is used to initialize gyro bias. Rotation matrix ${\hat{R}}_{w}^{b}$ is computed by aligning gravity in frame *b*, which is the mean static accelerometer data, with gravity in frame *w*. This initialization procedure is a rough one since accelerometer bias has not been eliminated, but its uncertainty can be modeled by the initial covariance matrix of the filter state.

**Algorithm 1** Automatic initialization procedure
Detect stationary sceneCounter=0**for** each image **do**  ${\mathrm{pix}}_{curr}=TrackFeatures\left(\right)$  **if** 0==Counter **then**    Counter++    ${\mathrm{pix}}_{prev}={\mathrm{pix}}_{curr}$    continue  **end if**  $\mathrm{diff}={\mathrm{pix}}_{curr}-{\mathrm{pix}}_{prev}$  **if** max(diff) is small enough **then**    Counter++  **else**    Counter=0    ${\mathrm{pix}}_{prev}={\mathrm{pix}}_{curr}$    continue  **end if**  **if** Counter is big enough **then**    break  **end if****end for**Initialize system stateSave stationary acc data in ${\mathrm{arry}}_{acc}$Save stationary gyro data in ${\mathrm{arry}}_{gyro}$$g_{b}=mean\left({\mathrm{arry}}_{acc}\right)$$g_{w}={\left[0,0,-9.8\right]}^{T}$${\hat{R}}_{w}^{b}=FromTwoVectors\left(g_{b},-g_{w}\right)$${\hat{b}}_{g}$ = $mean({\mathrm{arry}}_{gyro})$${\hat{v}}^{w}=0;{\hat{p}}^{w}=0;{\hat{b}}_{a}=0$;


### 6.3. Refined Feature Triangulation Mechanism

In Mourikis and Roumeliotis’s work [1], features are triangulated only if they are no longer being tracked; however, we found that this mechanism does not perform well, especially when using cheap IMUs. To conduct frequent and effective measurement updates, which is crucial to correct biased IMU propagation, a maximum feature tracking length is set. This means each feature would be triangulated when it has been tracked for a certain number of frames, even if it is still being tracked. In the latter situation, the current observation would not be used in triangulation.

Generally, features that failed in triangulation would be discarded directly. While the proposed mechanism is that if a feature fails in triangulation while it is still being tracked, it will have another chance to triangulate when the next image is coming. This mechanism improves the performance when the camera is moving slowly, where features in adjacent images exhibit a small parallax that would easily result in triangulation failure.

## 7. Experimental Results

The public dataset EuRoC [33] was used to evaluate the performance of the proposed VIO. It includes 11 sequences that were collected by a UAV in three different scenes. One is a machine hall and the other two are rooms equipped with motion capture systems and different manual layout arrangements. The extrinsic and intrinsic parameters of sensors are carefully calibrated, and ground truths of UAV poses are provided. It is one of the widely used benchmarks for evaluating algorithms of different configurations, including monocular-visual, stereo-visual and monocular/stereo-visual-inertial setups. All of the experiments below were performed on an Ubuntu 16.04 virtual machine powered by MacBook Pro Mid 2015 assigned with two core and 8 GB RAM. Our implementation is a real-time algorithm based on ROS nodelet [41].

**Figure 5:**
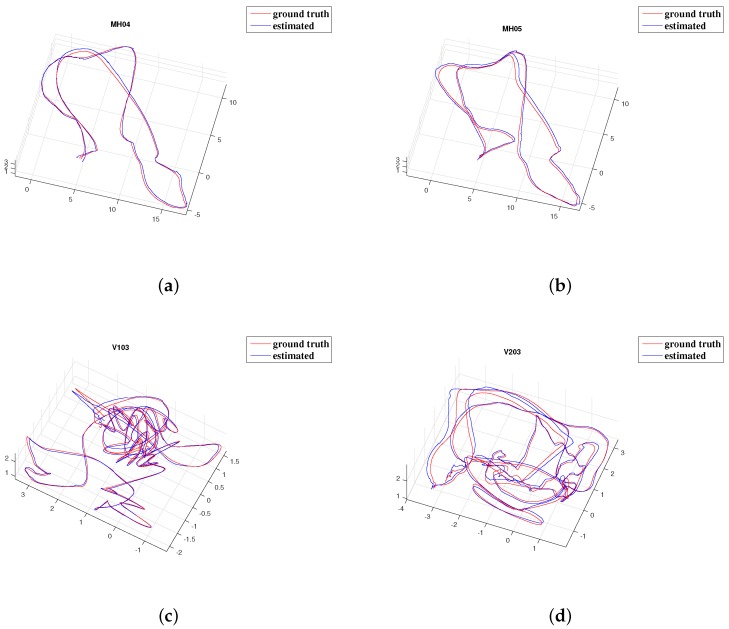
Results of 4 EuRoC sequences classified as “difficult”. Estimated trajectories are aligned with ground truths by a 6-DOF $Sim\left(3\right)$ transformation (without scale). (**a**) MH_04_difficult. (**b**) MH_05_difficult. (**c**) V1_03_difficult. (**d**) V2_03_difficult.

The estimated trajectories and corresponding ground truths are shown in Figure 5. Estimated trajectories are aligned with ground truths by a 6-DOF $Sim\left(3\right)$ transformation without adjusting the scale [42].

### 7.1. Front-End Improvement

**Figure 6:**
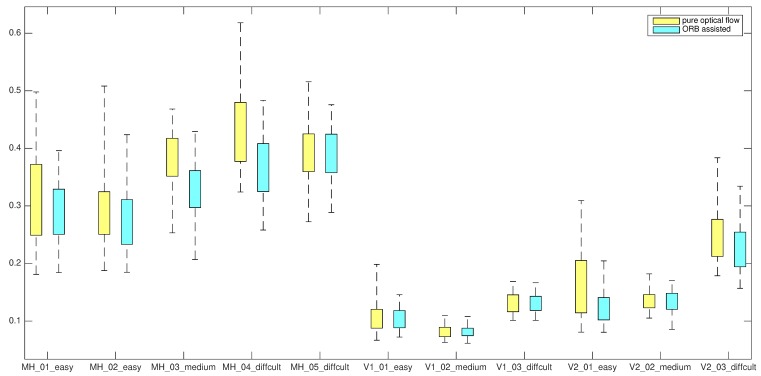
Boxplot summary of experimental results in terms of translation root-mean-square errors (RMSEs) of estimated trajectories. As can be seen, with ORB descriptor assistance the estimation is generally of higher precision, reflected in the lower position and narrower height of the corresponding box’s range for most sequences.

For implementations of front-ends with and without ORB descriptor assistance, we run each EuRoC sequence 50 times. A boxplot summary is shown in Figure 6. The corresponding means and standard deviations are listed in Table 1.

After adding ORB descriptor assistance, the estimator performs better in most sequences, since boxes became narrow and their position lower in Figure 6. The statistics in Table 1 give a numerical display of the results. Obvious improvement can be observed in seven sequences. In the other four sequences, performance are similar with or without ORB descriptor assistance. This may be due to the small quantity of outliers of optical flow tracking in these sequences.

We also analyzed the processing time of the proposed ORB descriptor-assisted outlier elimination procedure. The maximum feature number is set as 150. The results are listed in Table 2.

As shown in Table 2, the proposed ORB descriptor-assisted outlier elimination procedure introduces little computation. The processing time varies among sequences, mostly due to the motion speed. Sequences with aggressive motion tend to take less processing time than those with slow motion since fewer features are tracked in the former case, and fewer ORB descriptor distances need to be calculated.

### 7.2. Comparison with MSCKF-MONO

We compare our proposed monocular MSCKF with the open-source monocular MSCKF implementation MSCKF-MONO [23]. MSCKF-MONO has a visual front-end based on optical flow and utilizes first-order approximation state transition equations in filtering. It also applies observability-constrained Kalman filter (OC-KF) [21] to fix the observability problem, which would fix the wrong observability properties and improve filter performance. Note that ours does not apply any similar techniques.

In our experiment, we removed the coarse initialization and forbid the reset module in MSCKF-MONO because for some reason, MSCKF-MONO did not work properly on nearly half of sequences under the original coarse initialization, and reset does not help if there is no stop during running. The initial state was assigned by noisy ground truth for both our algorithm and MSCKF-MONO in this experiment. To make a fair comparison, we tried to run with same setup for common parameters in both algorithms, such as noise densities for sensors measurement, sliding window size, and maximum or minimum track lengths for features. However, MSCKF-MONO barely worked in any sequences under a similar setup as ours. This is mainly due to the different state transition model and visual front-end implementations. As we explored further and could not find a setup which generally performed better than the original setup for MSCKF-MONO, we left the original parameters unaltered. The comparison results are listed in Table 3.

The results show that the proposed monocular MSCKF is far more accurate than MSCKF-MONO. We claimed that this is due to a more accurate state transition model and a robust visual front-end.

### 7.3. Comparison with the State-Of-The-Art

The results of proposed VIO algorithm are compared with several state-of-the-art open-source monocular VIOs using the EuRoC dataset, including OKVIS [6], ROVIO [5], and VINS-MONO [2]. To make a fair comparison between pure VIOs, we turned off the closure detection in VINS-MONO. The proposed VIO automatically selects stationary IMU data to initialize the rotation and gyro bias at the beginning of every sequence, while other states are initialized as zeros. In addition, a unique parameter configuration is applied in all sequences. Results are listed in Table 4.

As shown above, the proposed VIO algorithm is comparable in accuracy to the state-of-the-art. Notice that VINS-MONO generally performs best out of all four algorithms, and the proposed algorithm has a similar performance in vicon rooms, which is due to good feature triangulation results in a limited area. In addition, the proposed algorithm and ROVIO perform better in V1_03 and V2_03 than others. There are aggressive motions in these two sequences that might result in tracking failure in the front-end; the proposed algorithm and ROVIO are filter-based methods that can utilize IMU measurements to propagate for a short period in this situation, while VINS-MONO and OKVIS sometimes fail and have to lean on relocalization in this circumstance. Notice that the machine hall is a relatively large-scale scenario [33], where triangulations in the proposed method mostly deal with points of large depth. This results in a relatively downgraded performance of the proposed method in the machine hall, even in sequences with mild motions.

### 7.4. Processing Time

As mentioned by Delmerico and Scaramuzza [17], the better performance of VINS-MONO is a trade-off requiring more computer resources than others. In contrast, the proposed method has a similar architecture to MSCKF-MONO, which is a light-weight solution. The average processing time of the visual front-end and EKF/optimization back-end of our implementation and the state-of-the-art are listed in Table 5.

The results show that, the proposed method has higher processing speed than the listed optimization-based methods. ROVIO is the fastest solution among all listed solutions, but as shown in Table 4, its precision is generally the worst. In proposed method, the visual front-end can process images at about 60 Hz. Notice that V2_03 is a little bit slower than others, because aggressive motions in this sequence result in a short feature tracking length, and thus, the front-end will take more time to extract new features. The EKF-based back-end run at more than 160Hz and the difference between each sequence is due to the difference in the number of features used in measurement updating. As can be concluded from Table 4 and Table 5, the proposed method is a VIO solution which has comparable precision and generally required less computation resources than the state-of-the-art.

## 8. Conclusions

In this paper, we first deduced a highly closed-form IMU error state transition equation from scratch. By using Hamilton’s notation of quaternion, we tried to eliminate notation ambiguity. We then managed to solve the integration terms left behind in the transition equation by introducing a two-sample fitting method to approximate the axis-angle, resulting in a fully linear closed-form formulation that is unbiased up to the fitting resolution. This formulation also has potential to incorporate IMU intrinsics into the filter state, since it is a linear function of IMU measurements. An automatic initialization procedure is developed and the feature triangulation mechanism is carefully refined. The ORB descriptor distance between Shi-Tomasi corner pairs was analyzed, and we found that there is a statistical difference in descriptor distances between matched and unmatched feature pairs. As outliers are sometimes fatal for filter-based VIOs, this inspired us to propose a visual front-end based on optical flow tracking and additionally, to use ORB descriptors to eliminate outliers. We implement a monocular VIO under the framework of MSCKF with proposed novelties.

Through a comparison between estimation results with and without the proposed outlier eliminating method, we demonstrate its effectiveness. Furthermore, an experiment was done to compare the proposed method with several state-of-the-art VIOs, both in terms of precision and computation. Results show that the proposed VIO is a visual inertial fusion solution with comparable precision to the state-of-the-ar but which demands less computation resources.

Future works include adding a robust initialization procedure adapting to versatile scenes and analyzing the point selection mechanism in detail.

## Figures and Tables

**Table 1:** Mean and standard deviation of RMSEs in Figure 6. For each sequence, the one with an obviously better performance is highlighted.

Sequence	MH_01		MH_02		MH_03		MH_04		MH_05		V1_01		V1_02		V1_03		V2_01		V2_02		V2_03	
	mean	std	mean	std	mean	std	mean	std	mean	std	mean	std	mean	std	mean	std	mean	std	mean	std	mean	std
pure optical flow	0.309	0.076	0.297	0.065	0.381	0.050	0.435	0.071	0.393	0.051	0.108	0.026	0.082	0.012	0.130	0.018	0.162	0.057	0.137	0.019	0.248	0.047
ORB assisted	**0.294**	**0.055**	**0.273**	**0.056**	**0.330**	**0.048**	**0.366**	**0.058**	0.391	0.046	**0.104**	**0.018**	0.082	0.010	0.131	0.017	**0.127**	**0.030**	0.134	0.019	**0.231**	**0.039**

**Table 2:** Mean of the processing time (ms) of the proposed ORB descriptor-assisted outlier elimination procedure for every image.

Sequence	MH_01	MH_02	MH_03	MH_04	MH_05	V1_01	V1_02	V1_03	V2_01	V2_02	V2_03
process time	1.3942	1.6480	1.3373	1.3983	1.0870	1.3297	1.0410	0.9574	1.2506	1.0525	0.7465

**Table 3:** Comparison results for proposed algorithm and MSCKF-MONO using the EuRoC dataset. The means of positioning RMSEs (m) of 10 runs for both algorithms are calculated.

	MH_01	MH_02	MH_03	MH_04	MH_05	V1_01	V1_02	V1_03	V2_01	V2_02	V2_03
MSCKF-MONO	1.015	0.534	0.427	2.102	0.968	0.169	0.275	1.551	0.281	0.341	×
Proposed	0.299	0.280	0.342	0.350	0.384	0.096	0.078	0.132	0.121	0.137	0.224

**Table 4:** Results of proposed and state-of-the-art VIOs using EuRoC dataset. Ten runs on each sequence and the means of positioning RMSEs (m) are calculated.

	MH_01	MH_02	MH_03	MH_04	MH_05	V1_01	V1_02	V1_03	V2_01	V2_02	V2_03
VINS-MONO	**0.159**	**0.182**	**0.199**	**0.350**	**0.313**	0.090	0.110	0.188	**0.089**	0.163	0.305
ROVIO	0.250	0.653	0.449	1.007	1.448	0.159	0.198	0.172	0.299	0.642	**0.190**
OKVIS	0.376	0.378	0.277	0.323	0.451	**0.087**	0.157	0.224	0.132	0.185	0.305
Proposed	0.289	0.258	0.331	0.394	0.423	0.117	**0.089**	**0.134**	0.097	**0.140**	0.211

**Table 5:** Average processing time (ms) and rate (Hz) of visual front-end and EKF/optimization back-end of our implementation and the state-of-the-art using the EuRoC dataset.

	Sequence	MH_01		MH_02		MH_03		MH_04		MH_05		V1_01		V1_02		V1_03		V2_01		V2_02		V2_03	
		Time	Rate	Time	Rate	Time	Rate	Time	Rate	Time	Rate	Time	Rate	Time	Rate	Time	Rate	Time	Rate	Time	Rate	Time	Rate
VINS-MONO	front-end	18.0	55	18.3	55	18.6	54	19.3	52	21.3	47	20.2	49	21.4	47	23.2	43	22.3	45	23.8	42	30.6	33
	back-end	50.2	20	50.9	20	50.1	20	50.1	20	53.0	19	53.1	19	45.9	22	37.9	26	54.4	18	48.3	21	33.4	30
ROVIO	front-end	2.0	505	1.9	526	2.0	497	2.1	476	2.0	490	1.9	538	2.0	508	2.1	481	2.0	503	2.0	510	2.0	478
	back-end	15.9	63	15.9	63	15.9	63	15.9	63	15.7	63	15.9	63	15.9	63	15.9	63	15.9	63	15.9	63	15.9	63
OKVIS	front-end	46.7	21	45.3	22	47.4	21	40.9	24	41.4	24	38.5	26	38.8	26	31.3	32	38.8	26	37.3	27	31.4	32
	back-end	39.8	25	39.4	25	39.9	25	32.1	31	33.1	30	30.6	33	25.5	39	19.2	52	29.6	34	27.9	36	18.0	56
Proposed	front-end	16.2	62	16.5	61	15.9	63	16.1	62	15.7	64	15.7	64	15.3	65	16.4	61	15.8	63	15.9	63	17.3	58
	back-end	5.5	182	5.9	169	6.1	164	5.5	181	6.0	166	5.7	174	5.4	185	4.9	203	5.7	176	5.6	178	4.6	218

## References

[1] Mourikis A.I., Roumeliotis S.I. A multi-state constraint Kalman filter for vision-aided inertial navigation. Proceedings of the IEEE International Conference on Robotics and Automation (ICRA).

[2] Qin T., Li P., Shen S. (2018). Vins-mono: A robust and versatile monocular visual-inertial state estimator. IEEE Trans. Robot..

[3] Von Stumberg L., Usenko V., Cremers D. Direct Sparse Visual-Inertial Odometry using Dynamic Marginalization. Proceedings of the IEEE International Conference on Robotics and Automation (ICRA).

[4] He Y., Zhao J., Guo Y., He W., Yuan K. (2018). PL-VIO: Tightly-Coupled Monocular Visual-Inertial Odometry Using Point and Line Features. Sensors.

[5] Bloesch M., Omari S., Hutter M., Siegwart R. Robust visual inertial odometry using a direct EKF-based approach. Proceedings of the 2015 IEEE/RSJ International Conference on Intelligent Robots and Systems (IROS).

[6] Leutenegger S., Lynen S., Bosse M., Siegwart R., Furgale P. (2015). Keyframe-based visual-inertial odometry using nonlinear optimization. Int. J. Robot. Res..

[7] Kümmerle R., Grisetti G., Strasdat H., Konolige K., Burgard W. g2o: A general framework for graph optimization. Proceedings of the IEEE International Conference on Robotics and Automation (ICRA).

[8] Kaess M., Johannsson H., Roberts R., Ila V., Leonard J.J., Dellaert F. (2012). iSAM2: Incremental smoothing and mapping using the Bayes tree. Int. J. Robot. Res..

[9] Liu H., Chen M., Zhang G., Bao H., Bao Y. ICE-BA: Incremental, Consistent and Efficient Bundle Adjustment for Visual-Inertial SLAM. Proceedings of the IEEE Conference on Computer Vision and Pattern Recognition (CVPR).

[10] Gui J., Gu D., Wang S., Hu H. (2015). A review of visual inertial odometry from filtering and optimisation perspectives. Adv. Robot..

[11] Aqel M.O., Marhaban M.H., Saripan M.I., Ismail N.B. (2016). Review of visual odometry: Types, approaches, challenges, and applications. SpringerPlus.

[12] Strasdat H., Montiel J., Davison A.J. Real-time monocular SLAM: Why filter?. Proceedings of the IEEE International Conference on Robotics and Automation (ICRA).

[13] Triggs B., McLauchlan P.F., Hartley R.I., Fitzgibbon A.W. (1999). Bundle adjustment—A modern synthesis. Proceedings of the 1999 International Workshop on Vision Algorithms.

[14] Lourakis M.I., Argyros A.A. (2009). SBA: A software package for generic sparse bundle adjustment. ACM Trans. Math. Softw. (TOMS).

[15] Hsiung J., Hsiao M., Westman E., Valencia R., Kaess M. Information Sparsification in Visual-Inertial Odometry. Proceedings of the IEEE/RSJ International Conference on Intelligent Robots and Systems (IROS).

[16] Agarwal S., Mierle K. Ceres Solver. http://ceres-solver.org.

[17] Delmerico J., Scaramuzza D. (2018). A Benchmark Comparison of Monocular Visual-Inertial Odometry Algorithms for Flying Robots. Memory.

[18] Eade E., Drummond T. Scalable monocular SLAM. Proceedings of the IEEE Computer Society Conference on Computer Vision and Pattern Recognition (CVPR).

[19] Li M., Mourikis A.I. Improving the accuracy of EKF-based visual-inertial odometry. Proceedings of the 2012 IEEE International Conference on Robotics and Automation (ICRA).

[20] Hesch J.A., Kottas D.G., Bowman S.L., Roumeliotis S.I. (2012). Observability-Constrained Vision-Aided Inertial Navigation.

[21] Huang G.P., Mourikis A.I., Roumeliotis S.I. (2010). Observability-based rules for designing consistent EKF SLAM estimators. Int. J. Robot. Res..

[22] Li M., Mourikis A.I. (2013). High-precision, consistent EKF-based visual-inertial odometry. Int. J. Robot. Res..

[23] Kostas Daniilidis R., Group of Prof Msckf-Mono. https://github.com/daniilidis-group/msckf_mono.

[24] Sun K., Mohta K., Pfrommer B., Watterson M., Liu S., Mulgaonkar Y., Taylor C.J., Kumar V. (2018). Robust stereo visual inertial odometry for fast autonomous flight. IEEE Robot. Autom. Lett..

[25] Zheng X., Moratto Z., Li M., Mourikis A.I. Photometric patch-based visual-inertial odometry. Proceedings of the IEEE International Conference on Robotics and Automation (ICRA).

[26] Zheng F., Tsai G., Zhang Z., Liu S., Chu C.C., Hu H. Trifo-VIO: Robust and Efficient Stereo Visual Inertial Odometry using Points and Lines. Proceedings of the IEEE/RSJ International Conference on Intelligent Robots and Systems (IROS).

[27] Trawny N., Roumeliotis S.I. (2005). Indirect Kalman Filter for 3D Attitude Estimation.

[28] Sommer H., Gilitschenski I., Bloesch M., Weiss S.M., Siegwart R., Nieto J. (2018). Why and How to Avoid the Flipped Quaternion Multiplication. Aerospace.

[29] Yang N., Wang R., Gao X., Cremers D. (2018). Challenges in monocular visual odometry: Photometric calibration, motion bias, and rolling shutter effect. IEEE Robot. Autom. Lett..

[30] Mur-Artal R., Tardós J.D. (2017). Visual-inertial monocular SLAM with map reuse. IEEE Robot. Autom. Lett..

[31] Bloesch M., Burri M., Omari S., Hutter M., Siegwart R. (2017). Iterated extended Kalman filter based visual-inertial odometry using direct photometric feedback. Int. J. Robot. Res..

[32] Rublee E., Rabaud V., Konolige K., Bradski G. ORB: An efficient alternative to SIFT or SURF. Proceedings of the IEEE International Conference on Computer Vision (ICCV).

[33] Burri M., Nikolic J., Gohl P., Schneider T., Rehder J., Omari S., Achtelik M.W., Siegwart R. (2016). The EuRoC micro aerial vehicle datasets. Int. J. Robot. Res..

[34] Titterton D., Weston J.L., Weston J. (2004). Strapdown Inertial Navigation Technology.

[35] Solà J. (2017). Quaternion Kinematics for the Error-State Kalman Filter.

[36] Qin Y. (2006). Inertial Navigation.

[37] Qin Y., Zhang H., Wang S. (2015). Kalman Filtering and Integrated Navigation Principles.

[38] Mur-Artal R., Tardós J.D. (2017). ORB-SLAM2: An Open-Source SLAM System for Monocular, Stereo, and RGB-D Cameras. IEEE Trans. Robot..

[39] Shi J., Tomasi C. (1993). Good Features to Track.

[40] Bouguet J.Y. (2001). Pyramidal implementation of the affine lucas kanade feature tracker description of the algorithm. Intel Corp..

[41] Quigley M., Conley K., Gerkey B., Faust J., Foote T., Leibs J., Wheeler R., Ng A.Y. ROS: An open-source Robot Operating System. Proceedings of the ICRA Workshop on Open Source Software.

[42] Umeyama S. (1991). Least-squares estimation of transformation parameters between two point patterns. IEEE Trans. Pattern Anal. Mach. Intell..

